# Leaky Bloch-like surface waves in the radiation-continuum for sensitivity enhanced biosensors via azimuthal interrogation

**DOI:** 10.1038/s41598-017-03515-0

**Published:** 2017-06-12

**Authors:** Vijay Koju, William M. Robertson

**Affiliations:** 1 Middle Tennessee State University, Computational Science Program, Murfreesboro, 37132 USA; 2 Middle Tennessee State University, Department of Physics and Astronomy, Murfreesboro, 37132 USA

## Abstract

Dielectric multilayer structures with a grating profile on the top-most layer adds an additional degree of freedom to the phase matching conditions for Bloch surface wave excitation. The conditions for Bloch surface wave coupling can be achieved by rotating both polar and azimuthal angles. The generation of Bloch surface waves as a function of azimuthal angle has similar characteristics to conventional grating coupled Bloch surface waves. However, azimuthally generated Bloch surface waves have enhanced angular sensitivity compared to conventional polar angle coupled modes, which makes them appropriate for detecting tiny variations in surface refractive index due to the addition of nano-particles such as protein molecules.

## Introduction

Bloch surface waves (BSW) are electromagnetic modes propagating at the interface of truncated dielectric multilayer structures and a homogeneous medium. The resonant generation of these modes via prism or grating coupling is an active field of current research. Following the prediction^1^ and experimental observation^2^ of BSWs in photonic crystals, these modes have been studied, both theoretically and experimentally, in various configurations^3–6^. The resulting strong field/energy localization at the surface layer and the evanescently extending field in the homogeneous medium are of interest in applications such as label-free biosensing based on enhanced diffraction^7–9^, surface-enhanced Raman spectroscopy^10, 11^, spectral and angular resonance shift^12–16^, fluorescence-based detection^17–20^, slow-light enhanced nonlinear effects^21, 22^, integrated optical circuits^23^, and optical slow-light devices and sensors^24, 25^. BSWs are evanescent in nature; they are perfectly bound non-radiative states that lie below the light line of the homogeneous layer material. However, it was recently shown that if the surface layer is periodically corrugated and the dielectric constant of the dielectric medium is real, positive, and large, it can support a leaky BSW^26^. Such leaky modes still lie below the light line of the homogeneous layer material but fall above that of the dielectric multilayer material. As a result, this leaky BSW is bound to the surface in the homogeneous region but is radiative into the dielectric multilayer. Moreover, under appropriate conditions, it is possible to excite photonic surface states inside the radiation continuum^27–29^. Although these states are radiative into the homogeneous medium, they can have a long lifetime assisted by destructive interference between different leakage channels. Such leaky-mode resonances, with moderate to infinitely high quality factor (Q) that confine freely propagating electromagnetic waves at a periodically modulated surface, are of interest in applications such as lossless mirrors^30^, high-performance optical filters^31^, label-free biosensors^32^, dielectric metasurfaces^33, 34^, dielectric-based optical magnetism^35^, and many others^36, 37^.

In this paper, we show through computational simulation that moderate-Q leaky BSWs on a dielectric multilayer surface with periodic corrugation can be used to significantly enhance the sensitivity of biosensors. To enhance the sensitivity we take advantage of the fact that the periodic corrugation of the surface layer allows us an additional degree of freedom over the azimuthal angle of the incident beam, which is not possible on a planar uncorrugated surface. In an experimental setup, this additional degree of freedom can be accessed by rotating the multilayer platform itself azimuthally. To our best knowledge however, little has been done in this regards^38, 39^. Previous related studies were done on surface plasmon polaritons (SPPs)—electromagnetic modes propagating at the interface of a metal and a dielectric medium—where the reflectivity is measured by fixing the azimuthal angle to a certain value followed by the conventional polar incident angle sweep. Here we propose a new technique of sensing using leaky BSWs, wherein we fix the polar incident angle to a specific value and then sweep over the azimuthal angle to fulfill the phase matching requirement to excite leaky BSWs. The advantages of this technique are twofold. First, it mitigates the requirement of a bulky prism to excite BSWs and thus affords the opportunity to engineer nanoscale lab-on-chip biosensors. Second, it can be used to make polarization independent biosensors, due to the fact that a linear grating profile facilitates polarization conversion^40–42^.

## Computational Method

**Figure 1:**
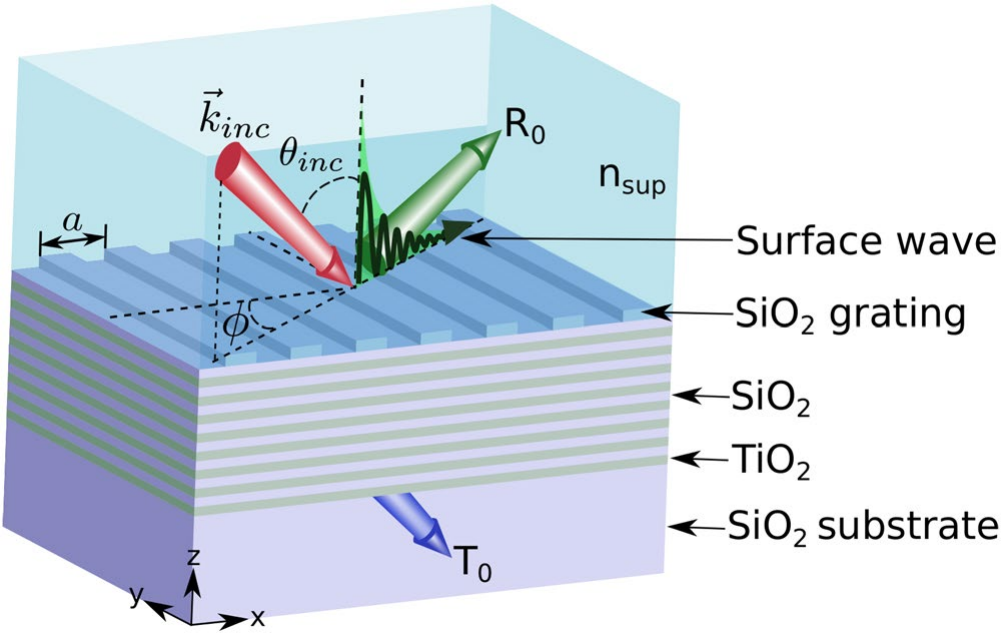
Schematic of a grating coupling technique to excite leaky Bloch surface waves on the surface of a dielectric multilayer.

A schematic of the computational setup considered in this study is shown in Fig. 1. We use a sixteen layered TiO_2_-SiO_2_ multilayer on a SiO_2_ substrate. The grating profile on the surface of the SiO_2_ layer is SiO_2_ as well. We consider the wavelength (*λ*) dependent refractive index of both TiO_2_^43^ and SiO_2_^44^ over the range of 0.43 *μ*m to 0.8 *μ*m as given by:

1 $${n}_{{{\rm{TiO}}}_{{\rm{2}}}}={(5.913+\frac{0.2441}{{\lambda }^{2}-0.0803})}^{\frac{1}{2}}$$

and

2 $${n}_{{{\rm{SiO}}}_{{\rm{2}}}}={(1+\frac{0.6962{\lambda }^{2}}{{\lambda }^{2}-{0.0684}^{2}}+\frac{0.4080{\lambda }^{2}}{{\lambda }^{2}-{0.1162}^{2}}+\frac{0.8975{\lambda }^{2}}{{\lambda }^{2}-{9.8962}^{2}})}^{\frac{1}{2}}$$

respectively. In the rest of the paper however, *λ* is given in nm. The index value of SiO_2_ films is well established. For TiO_2_ the presence of voids can reduce the index from that predicted; however, with careful fabrication^45^, the values used here are valid. There is ample evidence in the experimental literature that such multilayers, including those with surface grating structures^2, 4, 12, 13, 20, 23^, can be readily fabricated.

The thicknesses of TiO_2_ and SiO_2_ layers are 126.13 nm and 205.41 nm respectively. The excitation and confinement of BSWs on the surface of one-dimensional (1D) photonic crystals are highly sensitive to the thickness of the surface defect layer due to the effects of multiple reflections from the periodic dielectric multilayer beneath^46^. For this reason, we set the thickness of the top SiO_2_ layer to 280.03 nm. The grating height is set to 70 nm with a fill factor of 0.5*a*, where *a* is the grating period set to 510 nm. The refractive index of the superstrate layer (*n*_sup_) considered in our study is 1.26–1.4. The polar incident angle (*θ*_inc_) is measured relative to the surface normal, while the azimuthal angle (*ϕ*) is measured with respect to the plane perpendicular to the grating profile.

We use an in-house three-dimensional (3D) scattering-matrix-based rigorous coupled wave analysis (SMRCWA) method to simulate the electric/magnetic field (TE-polarized) distribution in the computational domain containing the multilayer structure, the SiO_2_ substrate, and the superstrate. The incident electric and magnetic fields are expressed in their Fourier expansion as

3 $${\bf{E}}(x,y,z)=\sum _{m=-\infty }^{\infty }\sum _{n=-\infty }^{\infty }{{\bf{S}}}_{{\rm{m}},{\rm{n}}}(z){e}^{-j({k}_{x,m}x+{k}_{y,n}y)}$$

4 $${\bf{H}}(x,y,z)=\sum _{m=-\infty }^{\infty }\sum _{n=-\infty }^{\infty }{{\bf{U}}}_{{\rm{m}},{\rm{n}}}(z){e}^{-j({k}_{{\rm{x}},{\rm{m}}}x+{k}_{{\rm{y}},{\rm{n}}}y)},$$

where

5 $${k}_{{\rm{x}},{\rm{m}}}={k}_{{\rm{x}},{\rm{inc}}}-\frac{2\pi m}{{{\rm{\Lambda }}}_{{\rm{x}}}},\quad \quad m=-\infty ,\ldots ,-2,-\mathrm{1,}\,\mathrm{0,}\,\mathrm{1,}\,2,\ldots ,\infty $$

6 $${k}_{{\rm{y}},{\rm{n}}}={k}_{y,\mathrm{inc}}-\frac{2\pi n}{{{\rm{\Lambda }}}_{{\rm{y}}}},\quad \quad n=-\infty ,\ldots ,-2,-\mathrm{1,}\,\mathrm{0,}\,\mathrm{1,}\,2,\ldots ,\infty \mathrm{.}$$

${k}_{{\rm{x}},{\rm{inc}}}=\frac{2\pi }{\lambda }{n}_{{\rm{\sup }}}\sin ({\theta }_{{\rm{inc}}})\cos (\varphi )$ and ${k}_{{\rm{y}},{\rm{inc}}}=\frac{2\pi }{\lambda }{n}_{{\rm{\sup }}}\sin ({\theta }_{{\rm{inc}}})\sin (\varphi )$ are the *x* and *y* components of **k**_inc_. Λ_x_ = *a* is the grating period in the *x* direction. The structure considered in this paper does not have any periodicity in the *y* direction. Thus Λ_y_ can be set to any non-zero value. For simplicity however, here we set Λ_y_ = *a* as well. **S**_m,n_(*z*) and **U**_m,n_(*z*) in Eqns (3) and (4) are the Fourier coefficients, which can be computed by solving Maxwell’s equations in Fourier space. The method is described in detail in Supplementary notes 1.

To verify the results obtained from the 3D SMRCWA method, we also do a 3D implementation of the structure in the commercial Finite Element Method software COMSOL Multiphysics. The results from both the methods are in good agreement. Moreover, the dispersion curves and the mode profiles were verified using Meep (an open source finite-difference time-domain (FDTD) software from MIT) as well.

## Results & Discussion

**Figure 2:**
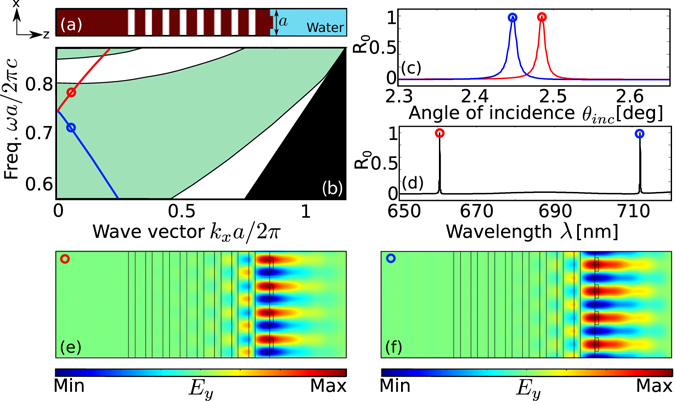
Leaky Bloch surface wave at *ϕ* = 0°. (**a**) TiO_2_-SiO_2_ multilayer with grating on the top layer. (**b**) Dispersion curves of the leaky BSWs supported by the structure. The green region is the radiative region (**c**,**d**) Reflectance of BSW as a function of incident angle and wavelength respectively. (**e**,**f**) Electric field profiles of BSW modes. The red and blue circles at the resonance peaks represent the reflectance at the corresponding circles in (**b**).

The proposed method consists in taking advantage of the surface grating profile, as illustrated in Fig. 1, to achieve sensing by selectively exciting BSWs via azimuthal interrogation. It is crucial to realize BSWs first in a setting where *ϕ* = 0°. A cross-section of a one period structure for this purpose is shown in Fig. 2(a). The surface grating serves as an input coupler that couples a plane wave (PW) into BSW mode. This BSW mode excitation is assisted by the constructive interference of PWs. At the correct incidence angle and groove spacing, a maximum coupling of the PWs to the BSW mode on the grating can be achieved, as summarized by Eqn. (7).

7 $${k}_{{\rm{BSW}}}=-{k}_{0}{n}_{{\rm{\sup }}}\sin ({\theta }_{{\rm{inc}}})+2\pi m/a,$$

where *k*_0_ = 2*π*/*λ* and *k*_BSW_ are the magnitudes of the free space wave vector and grating BSW wave vector respectively, *n*_sup_ is the refractive index of the superstrate, *θ*_inc_ is the incident angle, *a* is the grating period, and *m* is an integer^47^.

To excite grating-coupled BSWs for a given incident wavelength, the grating period *a* is chosen such that there is at least one angle that satisfies Eqn. (7), which leads to $\lambda /a < {k}_{{\rm{BSW}}}/{k}_{0}+1$. In this paper, we choose *m* = 1 and *θ*_inc_ > 0, such that $2\pi /a > {k}_{{\rm{BSW}}}$. Given that ${k}_{BSW} > {k}_{0}$, we get $\lambda /a/ > {k}_{{\rm{BSW}}}/{k}_{0} > 1$. Therefore, the range of appropriate grating periods can be summarized as ${k}_{{\rm{BSW}}}/{k}_{0} < \lambda /a < {k}_{{\rm{BSW}}}/{k}_{0}+1$.

The surface mode band structures of BSW modes for the structure are shown in Fig. 2(b). By terminating the surface layer with an additional thickness to act as a defect, we can create a platform for exciting BSWs. By etching a grating profile on top of it, we can mitigate the necessity of a prism to excite them. Periodicity on the surface plays an important role on these modes. The evanescent fields in the superstrate layer, in the presence of periodicity, can have wavevectors *k*_x_ in the reciprocal lattice that are integer multiples of 2*π*/*a*, resulting in BSW resonances. We observe two distinct BSW modes, highlighted in red and blue, in Fig. 2(b). Reflectivity curves of these modes, as a function of angle of incidence and wavelength, are shown in Fig. 2(c,d) respectively, where the colored tips correspond to their respective colored circle marks in Fig. 2(b). As can be seen in these figures, these modes have sharp resonance features at their excitation both as a function of angle and wavelength. Figure 2(e,f) show the field profile *E*_y_ of the BSW modes at the red (*λ* = 660 nm) and blue (*λ* = 710 nm) circles in Fig. 2(b) at ${\theta }_{{\rm{inc}}}\approx {2.5}^{\circ }$. At these surface-parallel wavevectors near ${k}_{{\rm{x}},{\rm{inc}}}\approx 0.04\times 2\pi /a$, the *E*_y_ field is highly confined to the surface giving rise to the BSW modes. We can also see a slight leakage in the superstrate layer. Time-domain field profiles of these modes are shown in Supplementary Videos 1 and 2. The direction of propagation of these modes in Fig. 2(d,e) are opposite however, which can be clearly seen in their time-domain profiles (see Supplementary Videos 1 and 2). This difference in the direction of propagation can be explained by the opposite sign slopes of the red and blue BSW modes in Fig. 2(b). These modes are different than conventional BSW modes as they exist in the radiative region. BSWs in general, only exist in the non-radiative region where they are perfectly bound to the surface. The BSW modes under consideration in this paper are not perfectly bound and are somewhat leaky. However, the quality factor ($Q=\omega \tau /2=(1/{Q}_{{\rm{r}}})+(1/{Q}_{{\rm{nr}}})$, where *Q*_r_ and *Q*_nr_ are the normalized radiative and non-radiative lifetimes due to leakage into free space) of these modes are high enough $(Q\approx 6000)$ that they can safely be used for practical applications in bio-sensing. The resonance lifetimes are extracted from the Fano features^28^ by fitting the reflectivity of the grating coupled multilayer structure to the thin-film reflectivity with the Fano features described by

8 $$f(\omega )=\frac{{Q}_{{\rm{r}}}^{-1}}{2i(1-\omega /{\omega }_{0})+{Q}_{{\rm{r}}}^{-1}+{Q}_{{\rm{nr}}}^{-1}}({r}_{{\rm{slab}}}-{t}_{{\rm{slab}}}),$$

where *ω*_0_ is the resonance frequency, and *r*_slab_ and *t*_slab_ are the reflection and transmission coefficients of a homogeneous slab, respectively. We further confirmed the quality factors of these modes using 2D FDTD simulations with point-dipole sources on the surface to perform harmonic analysis to compute the lifetime *τ* and *Q* of these resonant modes. The FDTD simulations were only performed for *ϕ* = 0°. The superstrate and substrate layers were terminated by perfectly matched layers, whereas the side boundaries were considered to be Floquet periodic boundaries. The mesh resolution was set to about 120 pixels per wavelength.

**Figure 3:**
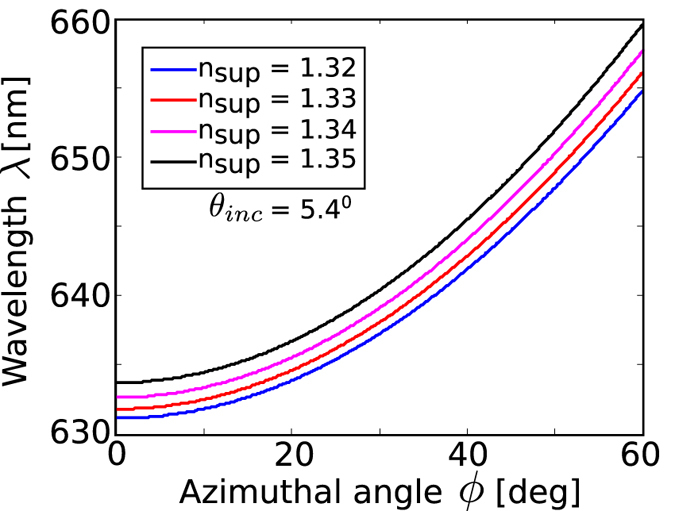
Azimuthal dispersion curves of BSWs for different values of superstrate refractive indices and *θ*_inc_ = 5.4°.

The previous paragraphs outline the conditions for coupling to BSWs by varying the polar angle, *θ*_inc_, with *ϕ* = 0°. We now examine the effect of altering the azimuthal angle *ϕ* to achieve BSW excitation. For this study we chose wavelengths in the vicinity of 632.8 nm corresponding to an experimental realization with a Helium-Neon laser. In this analysis the polar angle of the incident light was set to *θ*_inc_ = 5.4°. This angle is slightly greater than the resonant angle for polar coupling *θ*_BSW_ = 5.2°. The parallel wave vector of the incident light, allowing for variable *ϕ*, is given by ${k}_{x,\mathrm{inc}}=\frac{2\pi }{\lambda }{n}_{{\rm{\sup }}}\,\sin ({\theta }_{{\rm{inc}}})\cos (\varphi )$. With *θ*_inc_ fixed, the coupling of incident light to BSWs is realized by altering *ϕ*. Resonant generation of BSWs occurs when *k*_x,inc_ = *k*_BSW_. Fig. 3 shows the surface mode coupling to BSWs as a function of azimuthal angle and wavelength for different values of superstrate refractive indices. This figure indicates directly how azimuthal angle sensing is achieved. For a fixed incident wavelength the azimuthal angle of coupling changes with superstrate refractive index. Similarly, at fixed azimuthal angle, the coupling wavelength alters with superstrate index.

The question of sensitivity of biosensors based on surface electromagnetic wave resonance - either surface plasmon polaritons^48–50^ or Bloch surface waves^12, 13, 16, 17^ - is an active research topic. For biosensing applications these sensors operate by detecting the change in dielectric loading at the surface of excitation due to reactions such as antibody-antigen binding of proteins or DNA-probe reactions^13^. However, because such reactions are widely variable, the use of sensitivity to index change of the analyte is used to compare different sensor designs. The Kretschmann and Otto prism configurations are the most widely used surface wave resonance sensors with angular sensitivity in the range of 50–100°/RIU and wavelength sensitivity (at 630 nm) in the range of 970 nm/RIU^51^. In general the grating coupling technique, although having an advantage of not requiring a voluminous prism, has suffered with lower sensitivity compared to prism coupling techniques.

However, all the studies on sensitivity analysis of grating coupling techniques have utilized only polar angle interrogation. Recently, Romanato *et al*.^39^ studied sensitivity enhancement in grating coupled surface plasmon resonance by azimuthal control. The authors search for an optimal value of the azimuthal angle, set to that value, and sweep over the polar *θ*_inc_ angle. In this paper, we take an opposite approach. First the *θ*_inc_ angle is set to an angle greater than *θ*_BSW_ for *ϕ* = 0°, then the azimuthal angle is swept over to excite azimuthal BSW at appropriate *ϕ*.

**Figure 4:**
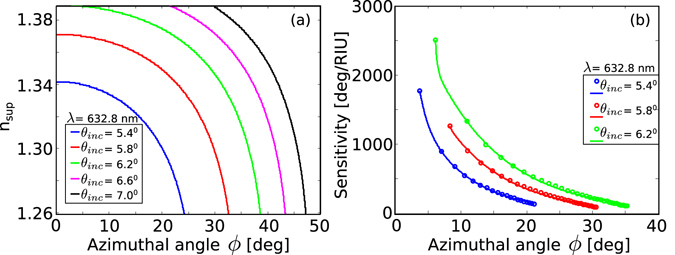
(**a**) BSW modes as a function of superstrate refractive index and azimuthal angle for different values of *θ*_inc_ at *λ* = 632.8 nm. (**b**) Azimuthal sensitivity of BSWs.

Figure 4(a) shows BSW modes supported by the structure as a function of superstrate refractive indices and azimuthal angle for different values of polar incident angles at the wavelength of 632.8 nm. We observe that for high values of *θ*_inc_, the azimuthal angular range for BSWs get wider. More importantly, at smaller azimuthal angles, the dispersion curves tend to flatten, i.e., for a small change in the superstrate refractive index, the change in the azimuthal angle is signifantly larger. This behavior has a crucial impact in increasing the azimuthal sensitivity of BSWs. The azimuthal sensitivity $({S}_{{n}_{{\rm{\sup }}},\varphi })$ is defined as

9 $${S}_{{n}_{{\rm{\sup }}},\varphi }=\frac{{\rm{\Delta }}\varphi }{{\rm{\Delta }}{n}_{{\rm{\sup }}}},$$

where, Δ*ϕ* is the change in the azimuthal angle and Δ*n*_sup_ is the change in the superstrate refractive index. The results for the azimuthal sensitivity given by Eqn. 9 are shown in Fig. 4(b). The sensitivity curves are computed from their respective colored BSW modes in Fig. 4(a). The improvement in the sensitivity can be clearly seen from the figure, with the azimuthal sensitivity as high as ~2500°/RIU. Higher sensitivity is especially useful for detecting tiny variations in the refractive index, as well as in detecting antibody-protein binding for disease detection. The sensitivity enhancement reported here is an order of magnitude higher compared to the previously reported grating coupled surface plasmon/BSW sensivities^5, 52–55^, which typically is in the range of 50–200°/RIU.

**Figure 5:**
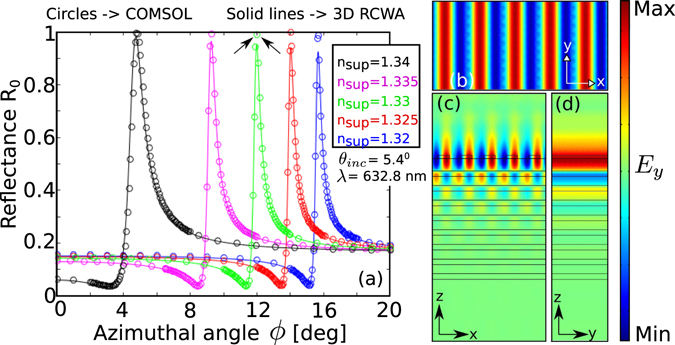
(**a**) Reflectivity curves as a function of azimuthal angle for different values of *n*_sup_. The wavelength (*λ*) and incident angle (*θ*) are fixed at 632.8 nm and 5.4° respectively. The results obtained using COMSOL (circles) and an in-house 3D RCWA code (solid lines) show good agreement. (**b**) x-y (**c**) x-z (**d**) y-z plane views of azimuthal BSW at the resonance peak indicated by the arrows in (**a**).

Figure 5(a) shows the reflectivity curves of the azimuthal BSWs for different values of the superstrate refractive indices. The solid curves are obtained using the 3D SMRCWA method, whereas the open circle curves are computed from COMSOL multiphysics. The results from both the numerical techniques are in excellent agreement with each other. We can observe from the figure that for a small change in the refractive index value $({\rm{\Delta }}{n}_{{\rm{\sup }}}=0.005)$, the azimuthal angular shift between the resonance peaks get larger at small azimuthal angles. Finally, the field profile of azimuthal BSW for the superstrate refractive index of 1.33 (water) is shown in Fig. 5(b–d). The corresponding resonance peak is indicated in Fig. 5(a) by the arrows. As in the case of conventional BSW (*ϕ* = 0°), the surface field intensity is highly amplified, and the mode is slightly leaky as well.

## Conclusion

In conclusion, we have studied a new way of exciting Bloch surface waves in dielectric multilayer structures with grating profile on the top-most layer via azimuthal interrogation. Fixing the polar incident angle to a value slighly higher than the BSW angle (for *ϕ* = 0° configuration), azimuthal BSWs can be excited by sweeping over the azimuthal angle. We show that as the refractive index of the superstrate layer increases, the azimuthal angular displacement between the BSW resonances increases as well. This change significantly increases the sensitivity of azimuthal BSWs. We report an order of magnitude higher sensitivity compared to the sensitivity of conventional BSWs.

## Electronic supplementary material

Supplementary note

Supplementary Video 1

Supplementary Video 2
