# Supplementary material for: Leaky Bloch-like surface waves in the radiation-continuum for sensitivity enhanced biosensors via azimuthal interrogation
Source: Sci Rep. 2017 Jun 12;7:3233. doi: 10.1038/s41598-017-03515-0 (PMC5468281; doi:10.1038/s41598-017-03515-0)
Supplement: Supplementary file 1 — Supplementary note [file 41598_2017_3515_MOESM1_ESM.pdf]

Supplemental material for

Leaky Bloch-like surface waves in the radiation-continuum for sensitivity enhanced biosensors via azimuthal interrogation

Vijay Koju[1,*] **and William M. Robertson**[1,2]

[1]Middle Tennessee State University, Computational Science Program, Murfreesboro, 37130, USA
[2]Middle Tennessee State University, Department of Physics and Astronomy, Murfreesboro, 37130, USA
[*]vk2g@mtmail.mtsu.edu

Introduction

Rigorous coupled wave analysis (RCWA)[1–7], as suggested by its name, is a more general formulation of Maxwell's equation for structures with material variation in x-y plane as well. The assumption here is that the medium may be inhomogeneous in x-y plane, but it must be uniform in the z-direction for each layer.

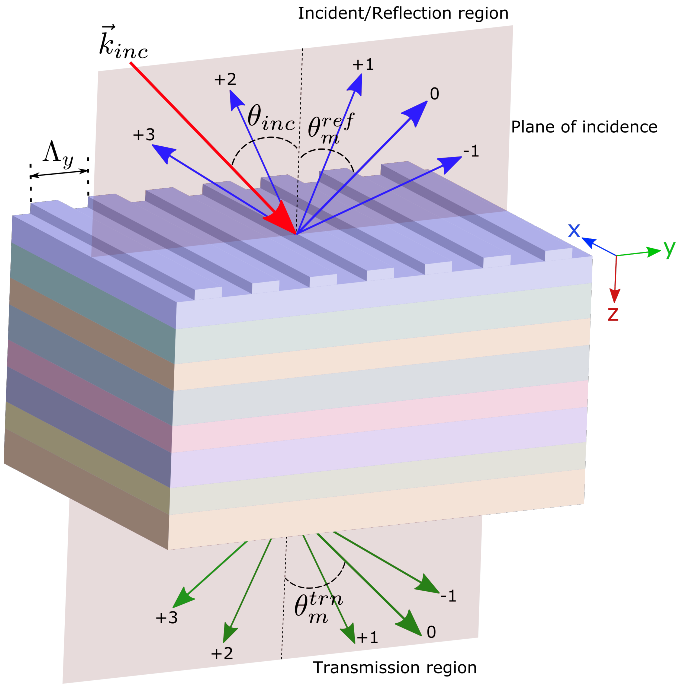

Figure 1. A schematic of a 1D multilayer structure with grating on the top layer.

We start with Maxwell's equations describing the fields inside a single linear, homogeneous, and isotropic layer of the device which are given below

$$\nabla \times \mathbf{E} = k_0 \mu_r \tilde{\mathbf{H}} \tag{1}$$

$$\nabla \times \tilde{\mathbf{H}} = k_0 \varepsilon_r \mathbf{E} \tag{2}$$

The term $\tilde{\mathbf{H}}$ is the normalized magnetic field which is equal to $-j\eta_0\mathbf{H}$, where $j = \sqrt{-1}$ and η_0 is the impedance of free space, k_0 is the free space wave number and is equal to $2\pi/\lambda_0$, where λ_0 is the free space wavelength, and μ_r and ε_r are relative

permeability and permittivity of the material respectively. Eqn. (1) and (2) can be expanded into a set of six coupled partial differential equations as follows

$$\frac{\partial E_z}{\partial y} - \frac{\partial E_y}{\partial z} = k_0 \mu_r \tilde{H}_x \tag{3}$$

$$\frac{\partial E_y}{\partial x} - \frac{\partial E_x}{\partial y} = k_0 \mu_r \tilde{H}_z \tag{4}$$

$$\frac{\partial E_x}{\partial z} - \frac{\partial E_z}{\partial x} = k_0 \mu_r \tilde{H}_y \tag{5}$$

$$\frac{\partial \tilde{H}_z}{\partial y} - \frac{\partial \tilde{H}_y}{\partial z} = k_0 \varepsilon_r E_x \tag{6}$$

$$\frac{\partial \tilde{H}_x}{\partial z} - \frac{\partial \tilde{H}_z}{\partial x} = k_0 \varepsilon_r E_y \tag{7}$$

$$\frac{\partial \tilde{H}_y}{\partial x} - \frac{\partial \tilde{H}_x}{\partial y} = k_0 \varepsilon_r E_z. \tag{8}$$

For RCWA, ε_r and μ_r are represented in terms of Fourier transforms along the x and y direction, the z-parameter remains analytical and unchanged.

$$\varepsilon_r(x,y) = \sum_{m=-\infty}^{\infty} \sum_{n=-\infty}^{\infty} a_{m,n} e^{j\left(\frac{2\pi mx}{\Lambda_x} + \frac{2\pi ny}{\Lambda_y}\right)} \tag{9}$$

$$\mu_r(x,y) = \sum_{m=-\infty}^{\infty} \sum_{n=-\infty}^{\infty} b_{m,n} e^{j\left(\frac{2\pi mx}{\Lambda_x} + \frac{2\pi ny}{\Lambda_y}\right)} \tag{10}$$

The coefficients $a_{m,n}$ and $b_{m,n}$ are given as

$$a_{m,n} = \frac{1}{\Lambda_x \Lambda_y} \int_{-\frac{\Lambda_x}{2}}^{\frac{\Lambda_x}{2}} \int_{-\frac{\Lambda_y}{2}}^{\frac{\Lambda_y}{2}} \varepsilon_r(x,y) e^{-j\left(\frac{2\pi mx}{\Lambda_x} + \frac{2\pi ny}{\Lambda_y}\right)} dxdy. \tag{11}$$

The terms Λ_x and Λ_y are the periods in x and y directions respectively.

$$b_{m,n} = \frac{1}{\Lambda_x \Lambda_y} \int_{-\frac{\Lambda_x}{2}}^{\frac{\Lambda_x}{2}} \int_{-\frac{\Lambda_y}{2}}^{\frac{\Lambda_y}{2}} \mu_r(x,y) e^{-j\left(\frac{2\pi mx}{\Lambda_x} + \frac{2\pi ny}{\Lambda_y}\right)} dxdy \tag{12}$$

The Fourier expansion of the fields are

$$E_x(x,y,z) = \sum_{m=-\infty}^{\infty} \sum_{n=-\infty}^{\infty} S_{x,m,n}(z) e^{-j(k_{x,m}x + k_{y,n}y)} \tag{13}$$

$$E_y(x,y,z) = \sum_{m=-\infty}^{\infty} \sum_{n=-\infty}^{\infty} S_{y,m,n}(z) e^{-j(k_{x,m}x + k_{y,n}y)} \tag{14}$$

$$E_z(x,y,z) = \sum_{m=-\infty}^{\infty} \sum_{n=-\infty}^{\infty} S_{z,m,n}(z) e^{-j(k_{x,m}x + k_{y,n}y)} \tag{15}$$

$$H_x(x,y,z) = \sum_{m=-\infty}^{\infty} \sum_{n=-\infty}^{\infty} U_{x,m,n}(z) e^{-j(k_{x,m}x + k_{y,n}y)} \tag{16}$$

$$H_y(x,y,z) = \sum_{m=-\infty}^{\infty} \sum_{n=-\infty}^{\infty} U_{y,m,n}(z) e^{-j(k_{x,m}x + k_{y,n}y)} \tag{17}$$

$$H_z(x,y,z) = \sum_{m=-\infty}^{\infty} \sum_{n=-\infty}^{\infty} U_{z,m,n}(z) e^{-j(k_{x,m}x + k_{y,n}y)}, \tag{18}$$

where

$$k_{x,m} = k_{x,inc} - \frac{2\pi m}{\Lambda_x}, \qquad m = -\infty, ..., -2, -1, 0, 1, 2, ..., \infty \tag{19}$$

$$k_{y,n} = k_{y,inc} - \frac{2\pi n}{\Lambda_y}, \qquad n = -\infty, ..., -2, -1, 0, 1, 2, ..., \infty \tag{20}$$

Substituting the field expressions in eqn. (3)-(8), and doing some algebraic manipulations, the Maxwell's equations in real space can be converted to the Maxwell's equations in semi-analytical Fourier space

$$-j\tilde{k}_{y,n} S_{z,m,n}(\tilde{z}) - \frac{d}{d\tilde{z}} S_{y,m,n}(\tilde{z}) = \sum_{q=-\infty}^{\infty} \sum_{r=-\infty}^{\infty} b_{m-q,n-r} U_{x,q,r}(\tilde{z}) \tag{21}$$

$$\frac{d}{d\tilde{z}} S_{x,m,n}(\tilde{z}) + j\tilde{k}_{x,m} S_{z,m,n}(\tilde{z}) = \sum_{q=-\infty}^{\infty} \sum_{r=-\infty}^{\infty} b_{m-q,n-r} U_{y,q,r}(\tilde{z}) \tag{22}$$

$$-j\tilde{k}_{x,m} S_{y,m,n}(\tilde{z}) + jk_{y,m} S_{z,m,n}(\tilde{z}) = \sum_{q=-\infty}^{\infty} \sum_{r=-\infty}^{\infty} b_{m-q,n-r} U_{z,q,r}(\tilde{z}) \tag{23}$$

$$-j\tilde{k}_{y,n} U_{z,m,n}(\tilde{z}) - \frac{d}{d\tilde{z}} U_{y,m,n}(\tilde{z}) = \sum_{q=-\infty}^{\infty} \sum_{r=-\infty}^{\infty} a_{m-q,n-r} S_{x,q,r}(\tilde{z}) \tag{24}$$

$$\frac{d}{d\tilde{z}} U_{x,m,n}(\tilde{z}) + j\tilde{k}_{x,m} U_{z,m,n}(\tilde{z}) = \sum_{q=-\infty}^{\infty} \sum_{r=-\infty}^{\infty} a_{m-q,n-r} S_{y,q,r}(\tilde{z}) \tag{25}$$

$$-j\tilde{k}_{x,m} U_{y,m,n}(\tilde{z}) + jk_{y,m} U_{z,m,n}(\tilde{z}) = \sum_{q=-\infty}^{\infty} \sum_{r=-\infty}^{\infty} a_{m-q,n-r} S_{z,q,r}(\tilde{z}). \tag{26}$$

Expanding each of these equations for every possible combination of m and n and putting them in matrix form, they can be compactly represented as

$$-j\mathbf{K}_y \mathbf{u}_z - \frac{d}{d\tilde{z}} \mathbf{u}_y = \varepsilon_r \mathbf{s}_x \tag{27}$$

$$\frac{d}{d\tilde{z}} \mathbf{u}_x + j\mathbf{K}_x \mathbf{u}_z = \varepsilon_r \mathbf{s}_y \tag{28}$$

$$\mathbf{K}_x\mathbf{u}_y - \mathbf{K}_y\mathbf{u}_x = j\varepsilon_r\mathbf{s}_z \tag{29}$$

$$-j\mathbf{K}_y\mathbf{s}_z - \frac{d}{d\tilde{z}}\mathbf{s}_y = \mu_r\mathbf{u}_x \tag{30}$$

$$\frac{d}{d\tilde{z}}\mathbf{s}_x + j\mathbf{K}_x\mathbf{s}_z = \mu_r\mathbf{u}_y \tag{31}$$

$$\mathbf{K}_x\mathbf{s}_y - \mathbf{K}_y\mathbf{s}_x = j\mu_r\mathbf{u}_z, \tag{32}$$

where

$$\mathbf{u}_x = \begin{bmatrix} U_{x,1,1} \\ U_{x,1,2} \\ \cdot \\ \cdot \\ \cdot \\ U_{x,M,N} \end{bmatrix} \quad \mathbf{u}_y = \begin{bmatrix} U_{y,1,1} \\ U_{y,1,2} \\ \cdot \\ \cdot \\ \cdot \\ U_{y,M,N} \end{bmatrix} \quad \mathbf{u}_z = \begin{bmatrix} U_{z,1,1} \\ U_{z,1,2} \\ \cdot \\ \cdot \\ \cdot \\ U_{z,M,N} \end{bmatrix} \quad \mathbf{s}_x = \begin{bmatrix} S_{x,1,1} \\ S_{x,1,2} \\ \cdot \\ \cdot \\ \cdot \\ S_{x,M,N} \end{bmatrix} \quad \mathbf{s}_y = \begin{bmatrix} S_{y,1,1} \\ S_{y,1,2} \\ \cdot \\ \cdot \\ \cdot \\ S_{y,M,N} \end{bmatrix} \quad \mathbf{s}_z = \begin{bmatrix} S_{z,1,1} \\ S_{z,1,2} \\ \cdot \\ \cdot \\ \cdot \\ S_{z,M,N} \end{bmatrix}$$

$$\mathbf{K}_x = \begin{pmatrix} \tilde{k}_{x,1,1} & & & \mathbf{0} \\ & \tilde{k}_{x,1,2} & & \\ & & \ddots & \\ \mathbf{0} & & & \tilde{k}_{x,M,N} \end{pmatrix} \qquad \mathbf{K}_y = \begin{pmatrix} \tilde{k}_{y,1,1} & & & \mathbf{0} \\ & \tilde{k}_{y,1,2} & & \\ & & \ddots & \\ \mathbf{0} & & & \tilde{k}_{y,M,N} \end{pmatrix}$$

$$\varepsilon_r = \begin{bmatrix} \text{Toeplitz Convolution matrix} \end{bmatrix} \qquad \mu_r = \begin{bmatrix} \text{Toeplitz Convolution matrix} \end{bmatrix}.$$

Eliminating the longitudinal components $\mathbf{s}_z$ and $\mathbf{u}_z$ by back-substitution and rearranging them, eqn. (27)-(32) can be further reduced down to

$$\frac{d}{d\tilde{z}}\mathbf{u}_x = \mathbf{K}_x\mu_r^{-1}\mathbf{K}_y\mathbf{s}_x + (\varepsilon_r - \mathbf{K}_x\mu_r^{-1}\mathbf{K}_x)\mathbf{s}_y \tag{33}$$

$$\frac{d}{d\tilde{z}}\mathbf{u}_y = (\mathbf{K}_y\mu_r^{-1}\mathbf{K}_y - \varepsilon_r)\mathbf{s}_x - \mathbf{K}_y\mu_r^{-1}\mathbf{K}_x\mathbf{s}_y \tag{34}$$

$$\frac{d}{d\tilde{z}}\mathbf{s}_x = \mathbf{K}_x\varepsilon_r^{-1}\mathbf{K}_y\mathbf{u}_x + (\mu_r - \mathbf{K}_x\varepsilon_r^{-1}\mathbf{K}_x)\mathbf{u}_y \tag{35}$$

$$\frac{d}{d\tilde{z}}\mathbf{s}_y = (\mathbf{K}_y\varepsilon_r^{-1}\mathbf{K}_y - \mu_r)\mathbf{u}_x - \mathbf{K}_y\varepsilon_r^{-1}\mathbf{K}_x\mathbf{u}_y \tag{36}$$

These equations can be compactly written in matrix form as follows

$$\frac{d}{d\tilde{z}}\begin{bmatrix} \mathbf{u}_x \\ \mathbf{u}_y \end{bmatrix} = \mathbf{Q}\begin{bmatrix} \mathbf{s}_x \\ \mathbf{s}_y \end{bmatrix} \tag{37}$$

$$\frac{d}{d\tilde{z}}\begin{bmatrix}\mathbf{s}_x\\ \mathbf{s}_y\end{bmatrix} = \mathbf{P}\begin{bmatrix}\mathbf{u}_x\\ \mathbf{u}_y\end{bmatrix}, \tag{38}$$

where

$$\mathbf{Q} = \begin{bmatrix}\mathbf{K}_x\mu_r^{-1}\mathbf{K}_y & \varepsilon_r - \mathbf{K}_x\mu_r^{-1}\mathbf{K}_x\\ \mathbf{K}_y\mu_r^{-1}\mathbf{K}_y - \varepsilon_r & -\mathbf{K}_y\mu_r^{-1}\mathbf{K}_x\end{bmatrix} \tag{39}$$

$$\mathbf{P} = \begin{bmatrix}\mathbf{K}_x\varepsilon_r^{-1}\mathbf{K}_y & \mu_r - \mathbf{K}_x\varepsilon_r^{-1}\mathbf{K}_x\\ \mathbf{K}_y\varepsilon_r^{-1}\mathbf{K}_y - \mu_r & -\mathbf{K}_y\varepsilon_r^{-1}\mathbf{K}_x\end{bmatrix} \tag{40}$$

Taking the derivative of eqn. (38) w.r.t. z' and then substituting eqn. (37) in the result, we get

$$\frac{d^2}{dz'^2}\begin{bmatrix}\mathbf{s}_x\\ \mathbf{s}_y\end{bmatrix} = \Omega^2\begin{bmatrix}\mathbf{s}_x\\ \mathbf{s}_y\end{bmatrix}, \tag{41}$$

where $\Omega^2 = \mathbf{PQ}$. Eqn. (41) is the second order wave equation in matrix form. It has a general solution of the form

$$\begin{bmatrix}\mathbf{s}_x(z')\\ \mathbf{s}_y(z')\end{bmatrix} = e^{-\Omega z'}\mathbf{s}^+(0) + e^{\Omega z'}\mathbf{s}^-(0). \tag{42}$$

The terms $\mathbf{s}^+(0)$, and $\mathbf{s}^-(0)$ are the initial values for this differential equation. The $\pm$ superscripts indicate whether they pertain to forward propagating waves (+) or backward propagating waves (-). The terms $e^{-\Omega z'}$ and $e^{\Omega z'}$ have a matrix as their exponents. These matrix exponentials can be computed using the eigen-vectors and eigen-values of the matrix Ω. Letting $\mathbf{W}$ and $\boldsymbol{\lambda^2}$ as the eigen-vector and eigen-value matrix of Ω^2, we can compute the matrix exponentials as

$$e^{-\Omega z'} = \mathbf{W}e^{-\lambda z'}\mathbf{W}^{-1} \tag{43}$$

$$e^{\Omega z'} = \mathbf{W}e^{\lambda z'}\mathbf{W}^{-1}, \tag{44}$$

where $e^{\pm\lambda z'} = diag(e^{\sqrt{\pm\lambda_1^2}z'}, e^{\sqrt{\pm\lambda_2^2}z'}, ..., e^{\sqrt{\pm\lambda_N^2}z'})$. Therefore, the solution can be written as

$$\begin{bmatrix}\mathbf{s}_x(z')\\ \mathbf{s}_y(z')\end{bmatrix} = \mathbf{W}e^{-\lambda z'}\mathbf{W}^{-1}\mathbf{s}^+(0) + \mathbf{W}e^{\lambda z'}\mathbf{W}^{-1}\mathbf{s}^-(0). \tag{45}$$

Finally, letting $\mathbf{W}^{-1}\mathbf{s}^+(0) \equiv \mathbf{c}^+$ and $\mathbf{W}^{-1}\mathbf{s}^-(0) \equiv \mathbf{c}^-$, which are column vectors of amplitude coefficients of the eigen-modes in the forward and backward directions respectively, we can rewrite eqn. (45) as

$$\begin{bmatrix}\mathbf{s}_x(z')\\ \mathbf{s}_y(z')\end{bmatrix} = \mathbf{W}e^{-\lambda z'}\mathbf{c}^+ + \mathbf{W}e^{\lambda z'}\mathbf{c}^- \tag{46}$$

The matrix $\mathbf{W} = \begin{bmatrix}1 & 0\\ 0 & 1\end{bmatrix} = I$ and $e^{\pm\lambda z'} = \begin{bmatrix}e^{\pm j\tilde{k}_z z'} & 0\\ 0 & e^{\pm j\tilde{k}_z z'}\end{bmatrix}$. The magnetic field has a similar solution given below

$$\begin{bmatrix}\mathbf{u}_x(z')\\ \mathbf{u}_y(z')\end{bmatrix} = -\mathbf{V}e^{-\lambda z'}\mathbf{c}^+ + \mathbf{V}e^{\lambda z'}\mathbf{c}^- \tag{47}$$

To compute $\mathbf{V}$, eqn. (47) is differentiated w.r.t. z'.

$$\frac{d}{dz'}\begin{bmatrix}\mathbf{u}_x(z')\\ \mathbf{u}_y(z')\end{bmatrix} = \mathbf{V}\lambda e^{-\lambda z'}\mathbf{c}^+ + \mathbf{V}\lambda e^{\lambda z'}\mathbf{c}^- \tag{48}$$

Then, substituting eqn. (46) into eqn. (37), and then comparing it to eqn. (48), we get $\mathbf{V} = \mathbf{QW}\lambda^{\mathbf{-1}}$. Combining the solutions of electric (eqn. (46)) and magnetic (eqn. (47)) fields into one matrix, we get,

$$\Psi(z') = \begin{bmatrix}\mathbf{s}_x(z')\\ \mathbf{s}_y(z')\\ \mathbf{u}_x(z')\\ \mathbf{u}_y(z')\end{bmatrix} = \begin{bmatrix}\mathbf{W} & \mathbf{W}\\ -\mathbf{V} & \mathbf{V}\end{bmatrix}\begin{bmatrix}e^{-\lambda z'} & \mathbf{0}\\ \mathbf{0} & e^{\lambda z'}\end{bmatrix}\begin{bmatrix}\mathbf{c}^+\\ \mathbf{c}^-\end{bmatrix} \tag{49}$$

Eqn. (49) represents electric and magnetic field in a layer of linear, homogeneous, and isotropic material.

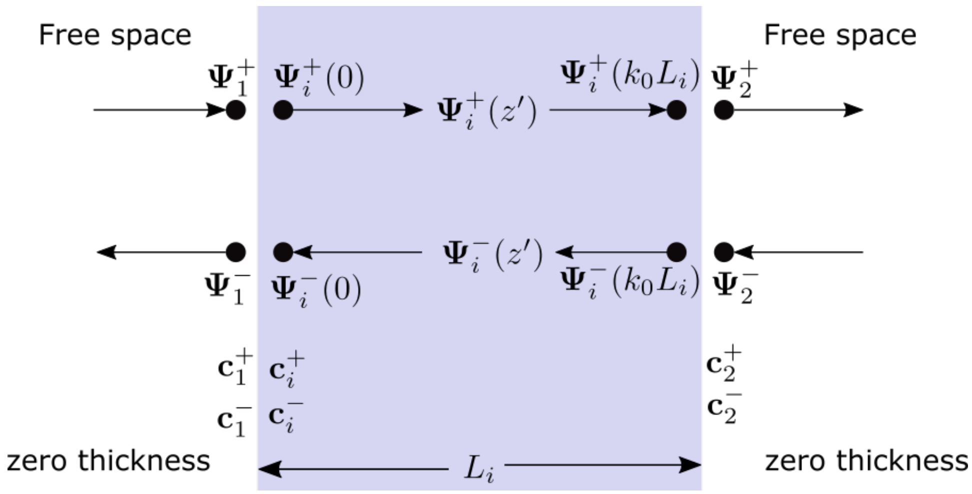

Figure 2. Field representations in the ith layer of a 1D multilayer.

0.0.1 Scattering matrix for a layer

A 1D multilayer device has a stack of multiple layers of different material. Thus, for a certain i^{th} layer in such a device, the solutions can be represented as

$$\Psi_i(z_i') = \begin{bmatrix} \mathbf{s}_{x,i}(z_i') \\ \mathbf{s}_{y,i}(z_i') \\ \mathbf{u}_{x,i}(z_i') \\ \mathbf{u}_{y,i}(z_i') \end{bmatrix} = \begin{bmatrix} \mathbf{W}_i & \mathbf{W}_i \\ -\mathbf{V}_i & \mathbf{V}_i \end{bmatrix} \begin{bmatrix} e^{-\lambda_i z_i'} & \mathbf{0} \\ \mathbf{0} & e^{\lambda_i z_i'} \end{bmatrix} \begin{bmatrix} \mathbf{c}_i^+ \\ \mathbf{c}_i^- \end{bmatrix} \tag{50}$$

Each layer has two interfaces with corresponding boundary conditions. Here, we considering each layer separately; thus, medium 1 (left region) and medium 2 (right region) do not strictly need to be layers of the 1D device in consideration. In fact, for the sake of numerical efficiency, we let both medium 1 and 2 be to free space of zero thicknesses. The boundary condition at the first (left) interface is

$$\Psi_1 = \Psi_i(0) \tag{51}$$

$$\begin{bmatrix} \mathbf{W}_1 & \mathbf{W}_1 \\ -\mathbf{V}_1 & \mathbf{V}_1 \end{bmatrix} \begin{bmatrix} \mathbf{c}_1^+ \\ \mathbf{c}_1^- \end{bmatrix} = \begin{bmatrix} \mathbf{W}_i & \mathbf{W}_i \\ -\mathbf{V}_i & \mathbf{V}_i \end{bmatrix} \begin{bmatrix} \mathbf{c}_i^+ \\ \mathbf{c}_i^- \end{bmatrix} \tag{52}$$

The boundary condition at the second (right) interface is

$$\Psi_i(k_0 L_i) = \Psi_2 \tag{53}$$

$$\begin{bmatrix} \mathbf{W}_i & \mathbf{W}_i \\ -\mathbf{V}_i & \mathbf{V}_i \end{bmatrix} \begin{bmatrix} e^{-\lambda_i k_0 L_i} & \mathbf{0} \\ \mathbf{0} & e^{\lambda_i k_0 L_i} \end{bmatrix} \begin{bmatrix} \mathbf{c}_i^+ \\ \mathbf{c}_i^- \end{bmatrix} = \begin{bmatrix} \mathbf{W}_2 & \mathbf{W}_2 \\ -\mathbf{V}_2 & \mathbf{V}_2 \end{bmatrix} \begin{bmatrix} \mathbf{c}_2^+ \\ \mathbf{c}_2^- \end{bmatrix} \tag{54}$$

After some manipulations of eqn. (52) and (54), we can reduce them to a system of the following form

$$\begin{bmatrix} \mathbf{c}_1^- \\ \mathbf{c}_2^+ \end{bmatrix} = \mathbf{S}^{(i)} \begin{bmatrix} \mathbf{c}_1^+ \\ \mathbf{c}_2^- \end{bmatrix}, \tag{55}$$

where

$$\mathbf{S}^{(i)} = \begin{bmatrix} \mathbf{S}_{11}^{(i)} & \mathbf{S}_{12}^{(i)} \\ \mathbf{S}_{21}^{(i)} & \mathbf{S}_{22}^{(i)} \end{bmatrix}. \tag{56}$$

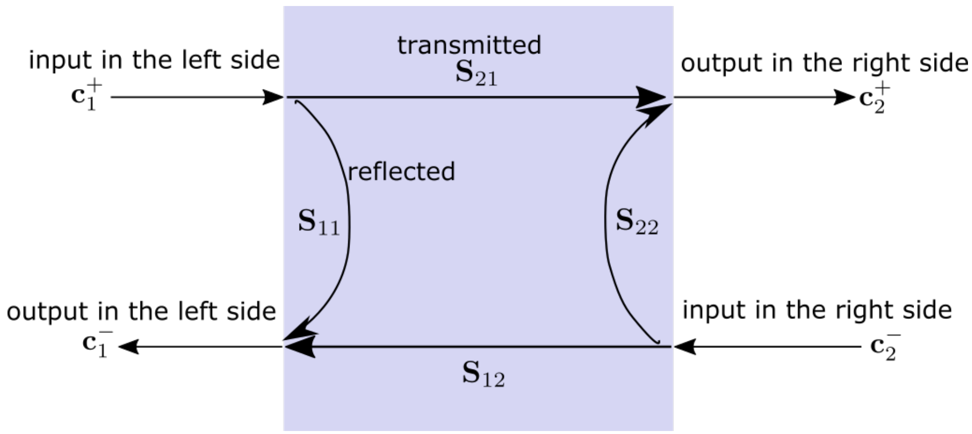

Figure 3. Representation of the scattering matrix of a single layer.

The element of matrix $\mathbf{S}^{(i)}$ are calculated as

$$\mathbf{S}_{11}^{(i)} = (\mathbf{A}_i - \mathbf{X}_i\mathbf{B}_i\mathbf{A}_i^{-1}\mathbf{X}_i\mathbf{B}_i)^{-1}(\mathbf{X}_i\mathbf{B}_i\mathbf{A}_i^{-1}\mathbf{X}_i\mathbf{A}_i - \mathbf{B}_i) \tag{57}$$

$$\mathbf{S}_{12}^{(i)} = (\mathbf{A}_i - \mathbf{X}_i\mathbf{B}_i\mathbf{A}_i^{-1}\mathbf{X}_i\mathbf{B}_i)^{-1}\mathbf{X}_i(\mathbf{A}_i - \mathbf{B}_i\mathbf{A}_i^{-1}\mathbf{B}_i) \tag{58}$$

$$\mathbf{S}_{21}^{(i)} = \mathbf{S}_{12}^{(i)} \tag{59}$$

$$\mathbf{S}_{22}^{(i)} = \mathbf{S}_{11}^{(i)} \tag{60}$$

The matrices $\mathbf{A}_i$, $\mathbf{B}_i$, and $\mathbf{X}_i$ are computed as

$$\mathbf{A}_i = \mathbf{W}_i^{-1}\mathbf{W}_0 + \mathbf{V}_i^{-1}\mathbf{V}_0 \tag{61}$$

$$\mathbf{B}_i = \mathbf{W}_i^{-1}\mathbf{W}_0 - \mathbf{V}_i^{-1}\mathbf{V}_0 \tag{62}$$

$$\mathbf{X}_i = e^{-\lambda_i k_0 L_i}. \tag{63}$$

The matrix $\mathbf{S}^{(i)}$ is the **scattering matrix** of the ith layer. It relates the input field to the output field. The elements $\mathbf{S}_{11}^{(i)}$, and $\mathbf{S}_{21}^{(i)}$ give reflection and transmission coefficients respectively. Because each layer is surrounded by free space in our formulation, the scattering matrices are symmetric. Thus only two of the matrix components have to be calculated for each layer.

0.0.2 Redheffer Star Product

In order to model a device with multiple layers, we need to combine multiple scattering matrices into a single scattering matrix. However, the scattering matrices cannot be combined directly by applying matrix multiplication. Also, the combined scattering matrix is not symmetric as the scattering matrix for a single layer so it becomes necessary to compute and store all four components of the combined scattering matrix. Two scattering matrices can be combined using the Redheffer star product[8,9]. The Redheffer star product of two scattering matrices $\mathbf{S}^{(A)} = \begin{bmatrix} \mathbf{S}_{11}^{(A)} & \mathbf{S}_{12}^{(A)} \\ \mathbf{S}_{21}^{(A)} & \mathbf{S}_{22}^{(A)} \end{bmatrix}$ and $\mathbf{S}^{(B)} = \begin{bmatrix} \mathbf{S}_{11}^{(B)} & \mathbf{S}_{12}^{(B)} \\ \mathbf{S}_{21}^{(B)} & \mathbf{S}_{22}^{(B)} \end{bmatrix}$ is defined as

$$\mathbf{S}^{(AB)} = \begin{bmatrix} \mathbf{S}_{11}^{(AB)} & \mathbf{S}_{12}^{(AB)} \\ \mathbf{S}_{21}^{(AB)} & \mathbf{S}_{22}^{(AB)} \end{bmatrix}, \tag{64}$$

where

$$\mathbf{S}_{11}^{(AB)} = \mathbf{S}_{11}^{(A)} + \mathbf{S}_{12}^{(A)}[\mathbf{I} - \mathbf{S}_{12}^{(B)}\mathbf{S}_{22}^{(A)}]^{-1}\mathbf{S}_{11}^{(B)}\mathbf{S}_{21}^{(A)} \tag{65}$$

$$\mathbf{S}_{12}^{(AB)} = \mathbf{S}_{12}^{(A)}[\mathbf{I} - \mathbf{S}_{12}^{(B)}\mathbf{S}_{22}^{(A)}]^{-1}\mathbf{S}_{12}^{(B)} \tag{66}$$

$$\mathbf{S}_{21}^{(AB)} = \mathbf{S}_{21}^{(B)}[\mathbf{I} - \mathbf{S}_{22}^{(A)}\mathbf{S}_{11}^{(B)}]^{-1}\mathbf{S}_{21}^{(A)} \tag{67}$$

$$\mathbf{S}_{22}^{(AB)} = \mathbf{S}_{22}^{(B)} + \mathbf{S}_{21}^{(B)}[\mathbf{I} - \mathbf{S}_{22}^{(A)}\mathbf{S}_{11}^{(B)}]^{-1}\mathbf{S}_{22}^{(A)}\mathbf{S}_{12}^{(B)} \tag{68}$$

0.0.3 Global Scattering matrix

The scattering matrix of a 1D multilayer with N layers can be computed by taking Redheffer star product of the scattering matrices of each layer.

$$\mathbf{S}^{(Device)} = \mathbf{S}^{(1)} \otimes \mathbf{S}^{(2)} \otimes \ldots \otimes \mathbf{S}^{(N-1)} \otimes \mathbf{S}^{(N)} \tag{69}$$

The multilayer device is surrounded by the reflection region and transmission region at its two ends. It is connected to these external materials by “connection” scattering matrices that have zero-thicknesses. The global scattering matrix finally combines all the scattering matrices into a single matrix as

$$\mathbf{S}^{(Global)} = \mathbf{S}^{(Ref)} \otimes \mathbf{S}^{(Device)} \otimes \mathbf{S}^{(Trn)} \tag{70}$$

The matrices $\mathbf{S}^{(Ref)}$ and $\mathbf{S}^{(Trn)}$ are the reflection and transmission region scattering matrices.

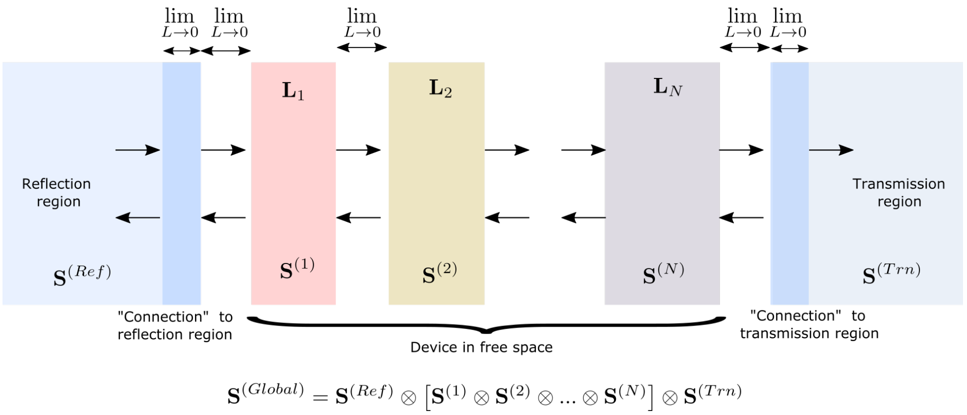

Figure 4. Representation of global scattering matrix.

$$\mathbf{S}^{(Ref)} = \begin{bmatrix} \mathbf{S}_{11}^{(Ref)} & \mathbf{S}_{12}^{(Ref)} \\ \mathbf{S}_{21}^{(Ref)} & \mathbf{S}_{22}^{(Ref)} \end{bmatrix}, \tag{71}$$

where

$$\mathbf{S}_{11}^{(Ref)} = -\mathbf{A}_{Ref}^{-1}\mathbf{B}_{Ref} \tag{72}$$

$$\mathbf{S}_{12}^{(Ref)} = 2\mathbf{A}_{Ref}^{-1} \tag{73}$$

$$\mathbf{S}_{21}^{(Ref)} = 0.5(\mathbf{A}_{Ref} - \mathbf{B}_{Ref}\mathbf{A}_{Ref}^{-1}\mathbf{B}_{Ref}) \tag{74}$$

$$\mathbf{S}_{22}^{(Ref)} = -\mathbf{B}_{Ref}\mathbf{A}_{Ref}^{-1}, \tag{75}$$

with

$$\mathbf{A}_{Ref} = \mathbf{W}_0^{-1}\mathbf{W}_{Ref} + \mathbf{V}_0^{-1}\mathbf{V}_{Ref} \tag{76}$$

$$\mathbf{B}_{Ref} = \mathbf{W}_0^{-1}\mathbf{W}_{Ref} - \mathbf{V}_0^{-1}\mathbf{V}_{Ref} \tag{77}$$

And

$$\mathbf{S}^{(Trn)} = \begin{bmatrix} \mathbf{S}_{11}^{(Trn)} & \mathbf{S}_{12}^{(Trn)} \\ \mathbf{S}_{21}^{(Trn)} & \mathbf{S}_{22}^{(Trn)} \end{bmatrix}, \tag{78}$$

where

$$\mathbf{S}_{11}^{(Trn)} = -\mathbf{B}_{Trn}\mathbf{A}_{Trn}^{-1} \tag{79}$$

$$\mathbf{S}_{12}^{(Trn)} = 0.5(\mathbf{A}_{Trn} - \mathbf{B}_{Trn}\mathbf{A}_{Trn}^{-1}\mathbf{B}_{Trn}) \tag{80}$$

$$\mathbf{S}_{21}^{(Trn)} = 2\mathbf{A}_{Trn}^{-1} \tag{81}$$

$$\mathbf{S}_{22}^{(Trn)} = -\mathbf{A}_{Trn}^{-1}\mathbf{B}_{Trn}, \tag{82}$$

with

$$\mathbf{A}_{Trn} = \mathbf{W}_0^{-1}\mathbf{W}_{Trn} + \mathbf{V}_0^{-1}\mathbf{V}_{Trn} \tag{83}$$

$$\mathbf{B}_{Trn} = \mathbf{W}_0^{-1}\mathbf{W}_{Trn} - \mathbf{V}_0^{-1}\mathbf{V}_{Trn}. \tag{84}$$

0.0.4 Calculating Transmitted and Reflected Electric Field

The global scattering matrix connects the input from the reflection region to the output at the transmission region

$$\begin{bmatrix} \mathbf{c}_{Ref} \\ \mathbf{c}_{Trn} \end{bmatrix} = \begin{bmatrix} \mathbf{S}_{11}^{(Global)} & \mathbf{S}_{12}^{(Global)} \\ \mathbf{S}_{21}^{(Global)} & \mathbf{S}_{22}^{(Global)} \end{bmatrix} \begin{bmatrix} \mathbf{c}_{inc} \\ \mathbf{0} \end{bmatrix}, \tag{85}$$

where

$$\mathbf{c}_{inc} = \mathbf{W}_{Ref}^{-1}\mathbf{s}_T^{inc} \tag{86}$$

The term $\mathbf{s}_T^{inc}$ is the electric field source defined as

$$\mathbf{s}_T^{inc} = \begin{bmatrix} p_x\delta_{0,pq} \\ p_y\delta_{0,pq} \end{bmatrix}, \tag{87}$$

where p_x and p_y are the x and y components of a unit amplitude polarization vector $\mathbf{P}$. The delta function ($\delta_{0,pq}$) is defined as $\delta_{0,pq} = [0,0,...,1,...,0,0]^T$. The coefficients for the reflected and transmitted fields can be computed from eqn. (85) as

$$\mathbf{c}_{Ref} = \mathbf{S_{11}}^{(Global)}\mathbf{c}_{inc} \tag{88}$$

$$\mathbf{c}_{Trn} = \mathbf{S_{21}}^{(Global)}\mathbf{c}_{inc} \tag{89}$$

The transverse components of the reflected and transmitted fields are then

$$\begin{bmatrix}\mathbf{r}_x \\ \mathbf{r}_y\end{bmatrix} = \mathbf{W}_{Ref}\mathbf{c}_{Ref} = \mathbf{W}_{Ref}\mathbf{S}_{11}^{(Global)}\mathbf{c}_{inc} \tag{90}$$

$$\begin{bmatrix}\mathbf{t}_x \\ \mathbf{t}_y\end{bmatrix} = \mathbf{W}_{Trn}\mathbf{c}_{Trn} = \mathbf{W}_{Trn}\mathbf{S}_{21}^{(Global)}\mathbf{c}_{inc}. \tag{91}$$

The longitudinal field components are calculated from the transverse components using the divergence equation and are given as

$$\mathbf{r}_z = -\mathbf{K}_{z,Ref}^{-1}(\mathbf{K}_x\mathbf{r}_x + \mathbf{K}_y\mathbf{r}_y), \tag{92}$$

and

$$\mathbf{t}_z = -\mathbf{K}_{z,Trn}^{-1}(\mathbf{K}_x\mathbf{t}_x + \mathbf{K}_y\mathbf{t}_y), \tag{93}$$

where $\mathbf{K}_{z,Ref} = -\left(\sqrt{\mu_{r,Ref}\varepsilon_{r,Ref}\mathbf{I} - \mathbf{K}_x^2 - \mathbf{K}_y^2}\right)^*$ and $\mathbf{K}_{z,Trn} = \left(\sqrt{\mu_{r,Trn}\varepsilon_{r,Trn}\mathbf{I} - \mathbf{K}_x^2 - \mathbf{K}_y^2}\right)^*$.

0.0.5 Calculating Diffraction Efficiencies

The diffraction efficiencies **R** and **T** are computed as

$$\mathbf{R} = \mathbb{R}\left[\frac{-\mathbf{K}_{z,Ref}}{k_{z,inc}}\right] \cdot |\mathbf{r}|^2, \tag{94}$$

and

$$\mathbf{T} = \mathbb{R}\left[\frac{\mu_{r,Ref}}{\mu_{r,Trn}}\frac{\mathbf{K}_{z,Trn}}{k_{z,inc}}\right] \cdot |\mathbf{t}|^2, \tag{95}$$

where

$$|\mathbf{r}|^2 = |\mathbf{r}_x|^2 + |\mathbf{r}_y|^2 + |\mathbf{r}_z|^2, \tag{96}$$

and

$$|\mathbf{t}|^2 = |\mathbf{t}_x|^2 + |\mathbf{t}_y|^2 + |\mathbf{t}_z|^2. \tag{97}$$

0.0.6 Calculating Overall Reflectance and Transmittance

Finally, the overall reflectance R and transmittance T can be calculated by adding all of the diffraction efficiencies as

$$R = \sum \mathbf{R}, \tag{98}$$

and

$$T = \sum \mathbf{T}. \tag{99}$$

References

1. M. G. Moharam, E. B. Grann, and D. A. Pommet, "Formulation for stable and efficient implementation of the rigorous coupled-wave analysis of binary gratings," *J. Opt. Soc. Am. A,* **12,** 1068 – 1076, (1995).
2. M. G. Moharam, E. B. Grann, D. A. Pommet, and T. K. Gaylord, "Stable implementation of the rigorous coupled-wave analysis for surface-relief gratings: enhanced transmittance matrix approach," *J. Opt. Soc. Am. A,* **12,** 1068 – 1076, (1995).

3. L. Li, "New formulation of the Fourier modal method for cross surface-relief gratings," *J. Opt. Soc. An. A,* **14,** 2758 – 2767, (1997).

4. T. K. Gaylord, and M. G. Moharam, "Analysis and applications of optical diffraction by gratings," *Proc. IEEE,* **73,** 894 – 937, (1985).

5. M. G. Moharam, and T. K. Gaylord, "Rigorous coupled-wave analysis of planar-grating diffraction," *J. Opt. Soc. Am.,* **71,** 811 – 818, (1981).

6. M. G. Moharam, and T. K. Gaylord, "Diffraction analysis of dielectric surface-relief gratings," *J. Opt. Soc. Am. A,* **72,** 1385 – 1392, (1982).

7. M. G. Moharam, and T. K. Gaylord, "Rigorous coupled-wave analysis of metallic surface-relief gratings," *J. Opt. Soc. Am. A,* **3,** 1780 – 1787, (1986)

8. R. C. Rumpf, "Improved formulation of scattering matrices for semi-analytical methods that is consistent with convention," *PIER B,* **35,** 241 – 261, (2011).

9. R. Redheffer, "Difference equations and functional equations in transmission-line theory," *Modern Mathematics for the Engineer,* **12,** 282 – 337, (1961).
