# Supplementary figures and images for: Leaky Bloch-like surface waves in the radiation-continuum for sensitivity enhanced biosensors via azimuthal interrogation
Source: Sci Rep. 2017 Jun 12;7:3233. doi: 10.1038/s41598-017-03515-0 (PMC5468281; doi:10.1038/s41598-017-03515-0)

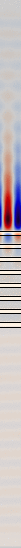

Supplement: Supplementary file 2 — Supplementary Video 1 [file 41598_2017_3515_MOESM2_ESM.gif]

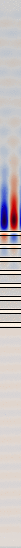

Supplement: Supplementary file 3 — Supplementary Video 2 [file 41598_2017_3515_MOESM3_ESM.gif]
